# Sodium-Iodate Injection Can Replicate Retinal Degenerative Disease Stages in Pigmented Mice and Rats: Non-Invasive Follow-Up Using OCT and ERG

**DOI:** 10.3390/ijms23062918

**Published:** 2022-03-08

**Authors:** Céline Koster, Koen T. van den Hurk, Jacoline B. ten Brink, Colby F. Lewallen, Boris V. Stanzel, Kapil Bharti, Arthur A. Bergen

**Affiliations:** 1 Department of Human Genetics, Section Ophthalmogenetics, Amsterdam University Medical Centers (AUMC), University of Amsterdam (UvA), Location AMC, Meibergdreef, 1105 AZ Amsterdam, The Netherlands; c.koster@amsterdamumc.nl (C.K.); k.t.vandenhurk@amsterdamumc.nl (K.T.v.d.H.); j.b.tenbrink@amsterdamumc.nl (J.B.t.B.); 2 Georgia Institute of Technology, G.W. Woodruff School of Mechanical Engineering, Atlanta, GA 30332, USA; colby.lewallen@gatech.edu; 3 Eye Clinic Sulzbach, Knappschaft Hospital Saar, 66280 Sulzbach/Saar, Germany; boris.stanzel@kksaar.de; 4 Department of Ophthalmology, University of Bonn, 53113 Bonn, Germany; 5 Ocular and Stem Cell Research Section, National Eye Institute, National Institutes of Health, Bethesda, MD 20892, USA; kapil.bharti@nih.gov; 6 Department of Ophthalmology, AUMC, UvA, Location AMC, Meibergdreef, 1105 AZ Amsterdam, The Netherlands

**Keywords:** retinal degeneration, retinal pigment epithelium, sodium iodate, mouse, rat, C57BL/6J, Brown Norway, rodent, OCT, ERG, macula degeneration

## Abstract

Purpose: The lack of suitable animal models for (dry) age-related macular degeneration (AMD) has hampered therapeutic research into the disease, so far. In this study, pigmented rats and mice were systematically injected with various doses of sodium iodate (SI). After injection, the retinal structure and visual function were non-invasively characterized over time to obtain in-depth data on the suitability of these models for studying experimental therapies for retinal degenerative diseases, such as dry AMD. Methods: SI was injected into the tail vein (i.v.) using a series of doses (0–70 mg/kg) in adolescent C57BL/6J mice and Brown Norway rats. The retinal structure and function were assessed non-invasively at baseline (day 1) and at several time points (1–3, 5, and 10-weeks) post-injection by scanning laser ophthalmoscopy (SLO), optical coherence tomography (OCT), and electroretinography (ERG). Results: After the SI injection, retinal degeneration in mice and rats yielded similar results. The lowest dose (10 mg/kg) resulted in non-detectable structural or functional effects. An injection with 20 mg/kg SI did not result in an evident retinal degeneration as judged from the OCT data. In contrast, the ERG responses were temporarily decreased but returned to baseline within two-weeks. Higher doses (30, 40, 50, and 70 mg/kg) resulted in moderate to severe structural RPE and retinal injury and decreased the ERG amplitudes, indicating visual impairment in both mice and rat strains. Conclusions: After the SI injections, we observed dose-dependent structural and functional pathological effects on the retinal pigment epithelium (RPE) and retina in the pigmented mouse and rat strains that were used in this study. Similar effects were observed in both species. In particular, a dose of 30 mg/kg seems to be suitable for future studies on developing experimental therapies. These relatively easily induced non-inherited models may serve as useful tools for evaluating novel therapies for RPE-related retinal degenerations, such as AMD.

## 1. Introduction

Age-related macular degeneration (AMD) is one of the major causes of visual impairment in the developed world [1,2,3,4,5]. The irreversible and non-treatable vision loss that is associated with AMD is a significant social burden, with a projected number of people with the disease around 288 million in 2040 worldwide [6]. Early detection and prevention are critical in increasing the likelihood of retaining good and functional vision [1].

Despite decades of research worldwide, the complex pathogenesis of AMD has not been fully elucidated yet [7]. In the early and intermediate stages, pathological hallmarks of AMD include the appearance of soft subretinal deposits and pigment alterations in the macular area of the retinal pigment epithelium (RPE). In advanced stages of the disease, “wet” and “dry” AMD can be distinguished. Wet AMD, also called neovascular AMD, is characterized by the abnormal growth of new (leaky) blood vessels through the RPE or into the neural retina of the eye. Dry AMD, also called geographic atrophy, is characterized by progressive atrophy of the RPE accompagnied by the degeneration of the adjacent neural retina and choriocapillaris. Clinically, “dry” AMD is the most common form, whereas the “wet” form affects roughly 10–15% of the patients, depending on ethnic group [8,9,10,11,12,13]. AMD onset and progression can, most likely, be delayed by refraining from smoking and dietary modifications. The wet form of AMD can be delayed by repeated anti-VEGF medication, but, to date, no cure for AMD exists once retinal damage has occurred [14].

While AMD affects multiple cell layers in the retina and also has a systemic component, the involvement of the retinal pigment epithelium (RPE) is key [15]. The RPE is a multifunctional single neuroepithelial cell layer that acts as a metabolic interface and retinal-blood barrier between the choroid and the neurosensory retina [16]. On the apical side, the photoreceptor cells line the RPE. On the basal side, the interposing Bruch’s membrane (BrM) separates the RPE from the choroidal microvasculature (choriocapillaris).

It is well known that multiple risk factors drive the pathogenesis of AMD, most prominently age, smoking, and genetic risk variation in multiple genes [5]. The latter include genes that are implicated in oxidative stress handling (*GPX4*), the complement system (*C9*, *CFI*, *CFH*), lipid metabolism (*APOE*, *ABCA1*, *PLTP*), and components of the extracellular matrix (*ARMS2-HTRA1, VTN*, *COL15/8A1*, *MMP9*, *MMP19*, *TIMP3*) [17,18,19,20,21,22,23,24].

Indeed, epidemiological, genetic, functional, physiological, and pathological studies support the hypothesis that, in particular, oxidative stress affecting the RPE is one of the main initial drivers of AMD [25,26,27,28,29,30,31]. Oxidative stress refers to elevated intracellular levels of reactive oxygen species (ROS) that cause damage to lipids, proteins, and DNA [32]. Local oxidative RPE stress is invoked by environmental and endogenous factors. There are two environmental risk factors for AMD (smoking and a high-fat diet) that increase the oxidative stress upon the retina and RPE [26,33,34].

Endogenous local oxidative stress factors on the RPE and retina include the daily renewal and digestion of (oxidized) photoreceptor outer segments, their intrinsically high metabolic rate, high dissolved oxygen concentration, and dependency on mitochondrial oxidative phosphorylation for proper function [35,36]. As a result, with aging, the RPE contains relatively high concentrations of oxidation-modified lipids and proteins, which probably contribute to the development of AMD over time [35,37,38]. Moreover, as people age, BrM’s permeability also decreases and a lipid wall builds up, hindering the reciprocal transport of nutrients and (oxidized) waste products between the RPE to the choroid [39,40]. Consequently, these oxidized molecules accumulate and the immune system is activated to clear them [41]. An excess of ROS can lead to nuclear and mitochondrial DNA damage, autophagy decay, and apoptosis of the RPE and photoreceptors [42].

Taking all the literature data together, there is strong evidence that oxidative stress leads to local RPE damage and eventually contributes AMD pathology over time [25,26,28,32,36,42,43].

Due to the complexity of AMD, the pathobiology of the disease has not yet been fully defined. Consequently, animal disease models of AMD have been reduced to more or less representing the critical disease symptoms. Therapeutic research into AMD has been hampered by the lack of suitable experimental models, both in vivo and in vitro [44]. Recently, a partial AMD cellular phenotype was replicated in vitro using an induced pluripotent stem cell-derived RPE culture harboring disease-associated *CFH* variants [45].

As described above, multiple mechanisms have been implicated in AMD, which are not fully represented in any genetically-modified animal model for monogenic retinal disease [45,46,47]. Some of these models represent, more or less, part of the AMD phenotype, such as the presence of drusenoid deposits [48,49,50,51]. As an alternative, chemically-induced models have been generated. The oxidizing chemical sodium iodate, NaIO_3_ (SI), causes necroptosis of the RPE cells and damage to its adjacent cell layers through oxidative stress-related processes [52,53,54]. As described above, this is a relevant mechanism for the development of dry AMD, especially geographic atrophy [55]. Consequently, such a model could be used for experimental therapeutic studies of retinal degenerative diseases, such as dry AMD. SI has been used in a wide variety of animals, injection methods, and doses. The SI-induced retinal degeneration model has been used to evaluate the transplantation of various experimental RPE-cell-based therapies [56,57,58]. However, comparing various published studies is often difficult due to notable differences in the experimental setup, such as the administration method, dosage, selected animals, and analyses time [59].

In this study, we further refined this model of SI-induced retinal degeneration to allow for reproducible induction by intravenous injection. We carefully followed the animals after the SI injection using non-invasive techniques. The post-injection retinal structure, morphology, and function were assessed at several time points in two different pigmented rodent species. This relatively easy and the rapidly induced non-inherited model may serve as a useful tool for evaluating novel therapies for retinal degenerations that are primarily caused by RPE failure.

## 2. Results

### 2.1. Sodium Iodate (SI) Injections in Mice and Rats

A total of twelve three-to-four-month-old C57BL/6J and eighteen eight-week-old Brown Norway rats received a single intravenous injection with sodium iodate (SI). Additionally, four mice and four rats received a single intravenous injection with 0.9% NaCl (saline; 0 mg/kg SI). This group served as a control group. A wide range of SI concentrations was used (0–70 mg/kg) to map a dose-dependent retinal degeneration and determine a potential model for experimental retinal tissue transplantations (see Supplementary Table S1). General health checks and weight progression graphs did not show any toxicologic effects up to 50 mg/kg (see Supplementary Figure S2). The animals were followed non-invasively after the injections using scanning laser ophthalmoscopy (SLO), optical coherence tomography (OCT), and electroretinography (ERG).

### 2.2. In Vivo Retinal Imaging Using an SLO-Based OCT System in Mice and Rats

**Figure 1:**
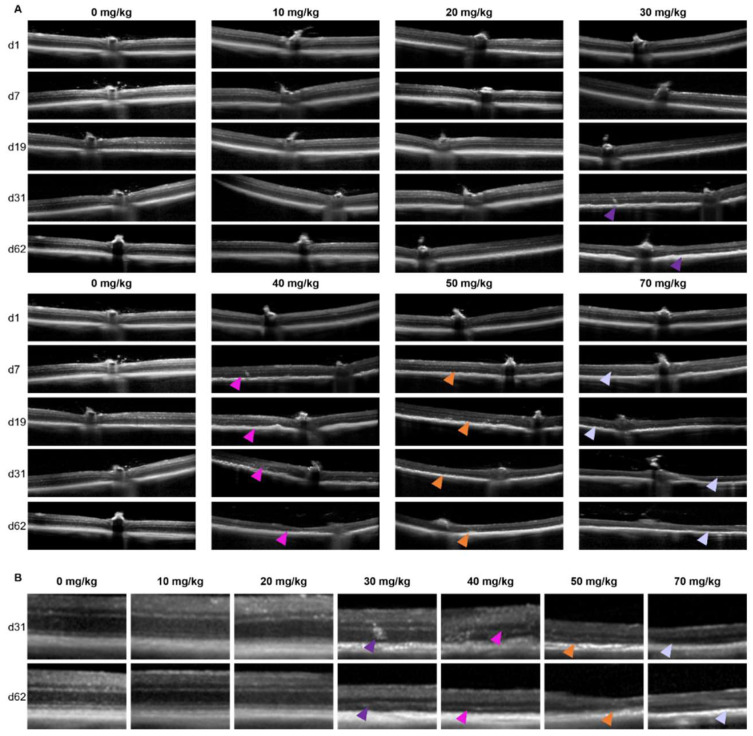
Representative serial OCT images of C57BL/6J mice of the central retina (**A**) and a magnification at the later timepoints (**B**) are shown. Serial scans are shown from single animals within the treatment group. In panel (**A**) the doses are horizontally shown in increasing concentration. The day of follow-up is shown vertically. No clear effects were observed for the 10 and 20 mg/kg groups compared to the control. Retinal degenerations was observed in all other groups. This is also better visible in panel (**B**), where the zoomed-in scans are shown per concentration SI (horizontally) and the later follow-up times (vertically). In the 30 mg/kg treatment group, retinal thinning started to show roughly 1 month post-injection (purple arrows). More drastic effects at the end of the experiments were observed for the 40 (pink arrows), 50 (orange arrows) and 70 mg/kg (lilac arrows) treatment groups. Quantification of the overall retinal thickness is shown in Figure 3.

**Figure 2:**
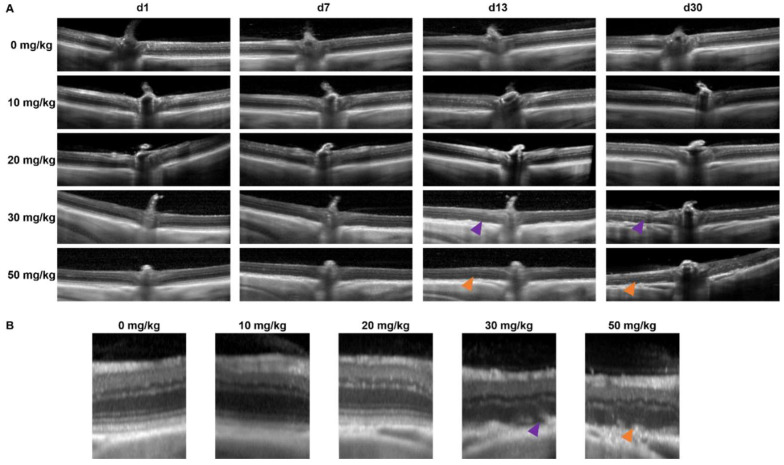
Representative serial OCT images of Brown Norway rats of the central retina (**A**) and a magnification at the later time points (**B**) are shown. Serial scans are shown from single animals within the treatment group. In panel (**A**) the doses are vertically shown in increasing concentration. The day of the follow-up is shown horizontally. No clear effects were observed for the 10 and 20 mg/kg groups compared to the control. Retinal degeneration is observed in both the 30 (purple arrows) and 50 mg/kg (orange arrows) groups at the later time points. Quantification of the overall retinal thickness is shown in Figure 3.

To determine the retinal morphology after the SI treatment, the mice and rats were followed over time non-invasively using an SLO-based spectral domain-OCT system. Representative OCT images are shown for mice in Figure 1 and rats in Figure 2. At the baseline (day 1: before injection), well-defined retinal layers were visible for all the animals. The retinal morphology of the control animals (0 mg/kg group) did not change over time. Similarly, minor to no changes were visible for the 10 and 20 mg/kg groups.

**Figure 3:**
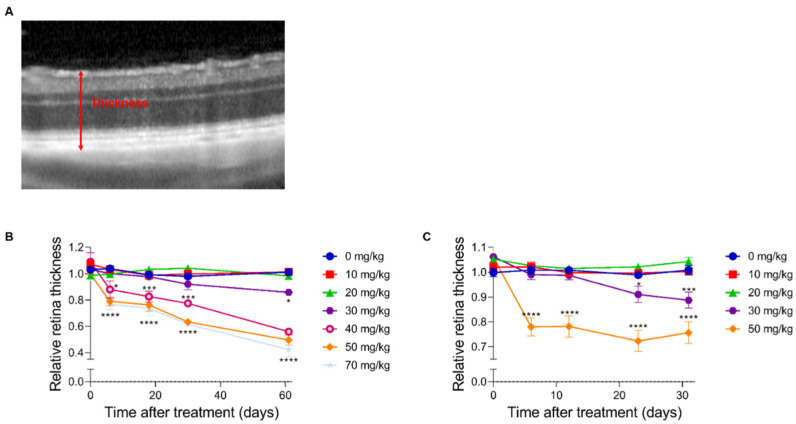
A quantitative analysis of the full retina thickness. The degeneration after SI injection was quantified in C57BL/6J mice (**B**) and Brown Norway rats (**C**) using the full thickness of the retina (red arrow) (**A**) (*n* = 4 per group). No significant effects were observed in both species for the 10 and 20 mg/kg treatment group compared to the control group (0 mg/kg). At two months post-injection, a thickness of ±90% of its original thickness was observed in the 30 mg/kg treatment group. More dramatic effects were observed in the higher treatment groups (40, 50, and 70 mg/kg). The degeneration started to show within a week post-injection. A relative thickness of 40–60% was observed two months post-injection. The results are presented as the mean ± the standard deviation. *: *p* ≤ 0.05, ***: *p* ≤ 0.001 and ****: *p* ≤ 0.0001.

In the 30 mg/kg groups, no retinal degeneration was visible on the OCT images within the first week. In contrast, at 13-days and later, RPE degeneration and disruption of the retinal layers started to show. The degeneration worsened over time without clear regeneration in the later stages. For the 40, 50, and 70 mg/kg groups, retinal thinning was visible within a week, and major retinal degeneration was visible from two-weeks onwards. The effects of the SI-treatments on the retinal structure were assessed using descriptive images (Figure 1 and Figure 2) as well as a quantitative approach (Figure 3). The retinal thickness was controlled and quantified consistently utilizing the manufacturer’s software and compared between the treatment groups. Our data and analyses suggest that in the 10 and 20 mg/kg groups, the thickness of the retina did not change up to the end of the experiment (62-days). The 30 mg/kg dose appeared to cause a moderate late-onset (two-weeks post-treatment) retinal degeneration. The 40, 50, and 70 mg/kg dose groups showed a relative extreme thinning of the retina already within one week. Thinning of the retina did not seem to be reversible over time for the duration of the experiment (62-days post-injection). Our data appears to show a preferential loss of the outer retinal layers. However, upon detailed analysis, we were not able to quantify this statistically. We obtained SLO images off all animals that were included in this study as well. However, we did not find any observable differences between the treatment groups (raw data available upon request).

At the end of the experiment, we could compare all groups, and we annotated the retinal thinning as “none” in the 0, 10, and 20 mg/kg groups, “intermediate” in the 30 mg/kg group and “high” in the 40, 50, and 70 mg/kg groups.

### 2.3. In Vivo Assessment of the Retinal Function Using ERG in Mice and Rats: A General Overview

**Figure 4:**
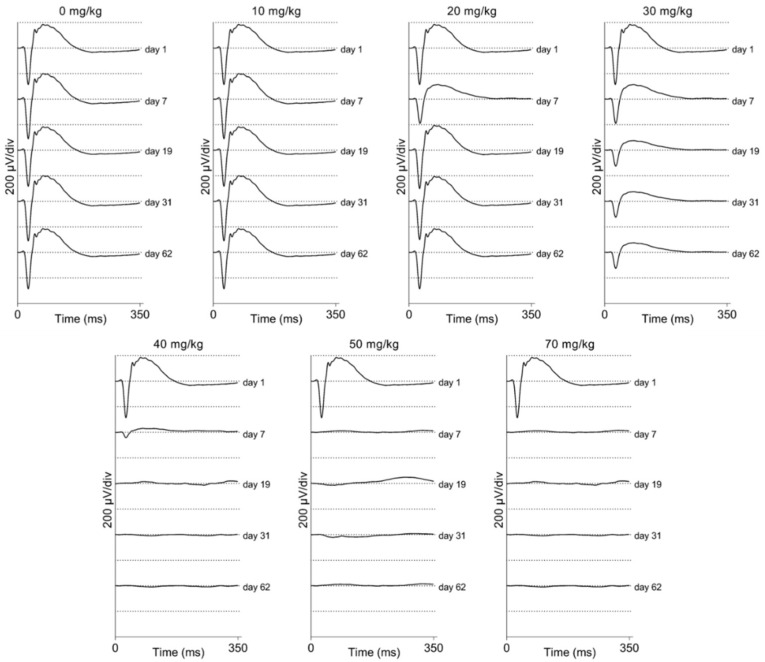
Averaged scotopic electroretinograms are shown for C57BL/6J mice (*n* = 4 per group). The averaged traces per treatment group and per day of follow-up are plotted. No difference can be observed between the 10 mg/kg group and the control group by eye. A temporary effect was seen for the 20 mg/kg group: a slightly decreased response was seen a week post-injection. A moderate effect was seen for the 30 mg/kg group: decreased responses were observed from seven-days post-injection onwards. This effect is larger later in time but seems stable after one-month post-injection. Direct and drastic effects were observed for higher treatment groups (40, 50, and 70 mg/kg). ERG responses were (almost) completely not recordable from one-week post-injection onwards. Similar effects were observed in ERG recordings from Brown Norway rats (Figure 5).

**Figure 5:**
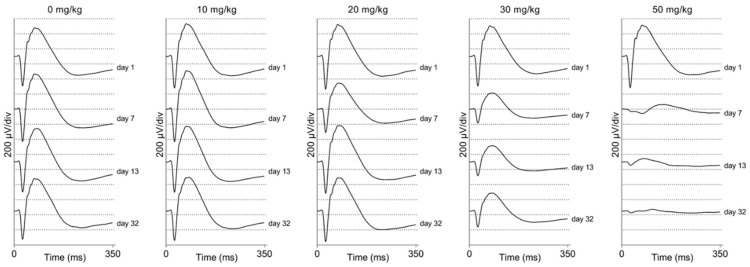
Averaged scotopic electroretinograms are shown for Brown Norway rats (*n* = 4 per group). The averaged traces per treatment group and per day of follow-up are plotted. No difference could be observed between the 10 mg/kg group and the control group by eye. A temporary effect was seen for the 20 mg/kg group: a slightly decreased response was seen a week post-injection. A moderate effect was seen for the 30 mg/kg group: decreased responses were observed from seven-days post-injection onwards. Direct and drastic effects were observed for the higher treatment group (50 mg/kg). The ERG responses were hardly recordable from one-week post-injection onwards. Similar effects were observed in the ERG recordings from C57BL/6J mice (Figure 4).

We performed electrophysiological studies in both the mice and rats to assess the visual function after SI treatment. We measured between one week and two months post-injection. Scotopic ERG traces are presented for C57BL/6J mice (Figure 4) and Brown Norway rats (Figure 5). It appears that the lower doses (0 and 10 mg/kg) did not have an obvious impact on the electrophysiological response of the eye. However, a 20 mg/kg dose seemed to cause a temporary effect around day seven post-injection. Doses that were equal and greater than 30 mg/kg caused a non-recoverable and immediate decrease in the response magnitude. The higher doses (40, 50, and 70 mg/kg) almost completely eliminated the recordable a- and b-waves, oscillatory potentials, and 9 Hz-flicker responses in both species. Thus, we observed a dose-dependent functional effect after SI treatment. No significant differences between control rats and mice ERG responses were observed (see Supplementary Figure S2).

### 2.4. Electroretinographic Recordings Discussed in More Detail in SI-Treated C57BL/6J Mice

**Figure 6:**
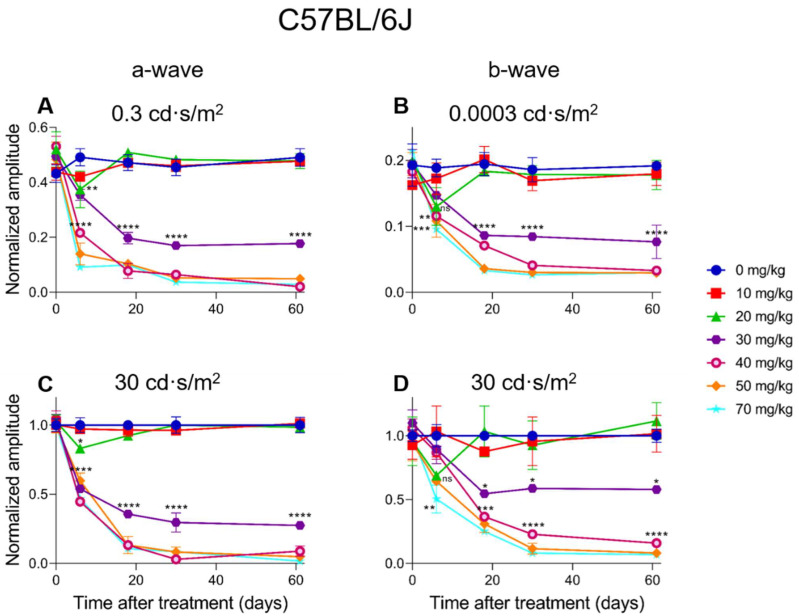
The normalized a- wave (**A**,**C**) and b-wave (**B**,**D**) amplitudes are plotted versus the time after treatment for all the treatment groups in C57BL/6J mice (*n* = 4 per group). The amplitudes are shown measured at 0.3 cd·s/m^2^ (**A**) and 30 cd·s/m^2^ (**C**) (a-wave) and 0.0003 cd·s/m^2^ (**B**) and 30 cd·s/m^2^ (**D**) (b-wave) including the standard deviations. No significant effect was observed for the 10 mg/kg group compared to the control. Although not significant for the b-wave, a temporary effect, and significant for the a-wave amplitude, was seen for the 20 mg/kg treatment group (**A**,**C**). This effect was not observed anymore later in the experiment. From seven-days post-injection onwards, significant decreased responses were observed for all the other treatment groups. The effects of the 30 mg/kg group was moderate and more prominently visible in the a-wave amplitude compared to the b-wave amplitude for both light intensities. Tremendous effects were seen for the higher treatment groups (40, 50, and 70 mg/kg). From three-weeks post-injection onwards, almost non-detectable a-wave amplitudes were observed and extremely decreased b-wave amplitudes. ns: not significant, *: *p* ≤ 0.05, **: *p* ≤ 0.01, ***: *p* ≤ 0.001, and ****: *p* ≤ 0.0001.

**Figure 7:**
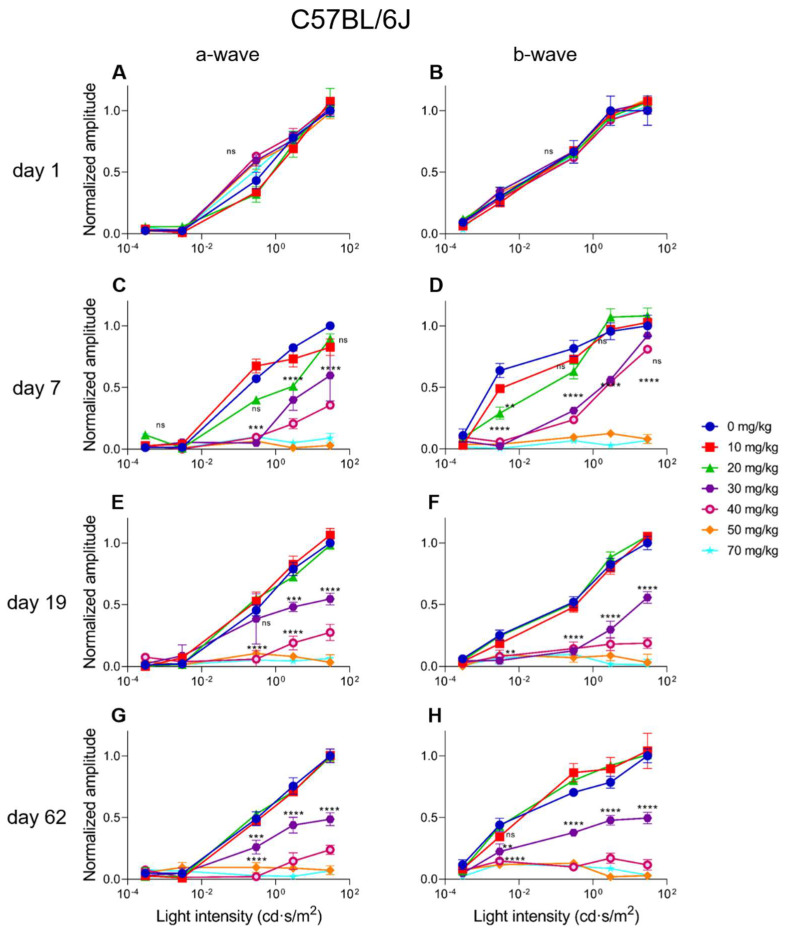
The normalized a-wave (**A**,**C**,**E**,**G**) and b-wave (**B**,**D**,**F**,**H**) amplitudes are plotted versus the light intensity for all the treatment groups in C57BL/6J mice (*n* = 4 per group). The amplitudes are shown ± the standard deviations. No significant differences can be observed at day 1 (baseline) between all the treatment groups. The treatment effects started to show from seven-days post-injection onwards. A slight effect was observed for the 20 mg/kg treatment group for both the a- and b-wave. The 30 and 40 mg/kg caused similar and moderate effects, and 50 and 70 mg/kg caused more extreme decreased amplitudes. From day 19 onwards, more long-term effects were visible. No significant differences were observed between the control, 10 mg/kg and 20 mg/kg groups. A significant and moderate effect was observed for the 30 mg/kg treatment group. Injection of 30 mg/kg SI caused a reduction of the a- and b-wave amplitudes of roughly 50% at least up to two-months post-injection. More extreme effects were observed for the higher doses (40, 50 and 70 mg/kg). Reduced responses to 20% to non-recordable ERG responses were observed in these groups. ns: not significant, **: *p* ≤ 0.01, ***: *p* ≤ 0.001, and ****: *p* ≤ 0.0001.

In C57BL/6J mice, a dose-dependent response for SI can be observed. A 10 mg/kg SI dose did not cause significant differences compared to the controls (see Figure 6 and Figure 7). A dose of 20 mg/kg caused a significant decrease for the a-wave amplitude one-week post-injection for both the low (0.3 cd·s/m^2^) and the high (30 cd·s/m^2^) light intensity (Figure 6A,C). Although a slight drop of the b-wave amplitude was seen in this treatment group for both the low (0.0003 cd·s/m^2^) and the high (30 cd·s/m^2^) light intensity, it was not significant (Figure 6B,D). The effect of SI on the a- and b-wave amplitudes were not seen later in the experiment. A higher dose of 30 mg/kg caused a more moderate and permanent effect on the ERG amplitudes. At this dose, not only the a-wave but also the b-wave amplitude was significantly decreased from three-weeks post-injection onwards. Indeed, a reduction of roughly 50% for both waves was observed. Even more extreme detrimental effects were observed for the higher doses (40, 50, and 70 mg/kg). From seven-days post-injection onwards, a- and b-wave amplitudes for low and high light intensities were significantly reduced. Reductions to <10% of the original amplitude values were reached and remained permanently low throughout the experiment. The effects of the intermediate (30 mg/kg) and high (40, 50, and 70 mg/kg) SI doses were observed from seven-days-post treatment for almost all the light intensities (see Figure 7A,D). From 19-days onwards, a moderate reduction was observed for the 30 mg/kg treatment group and a more extreme effect for the 40, 50, and 70 mg/kg treatment groups (Figure 7B,C,E,F). Finally, for C57BL/6J mice, Flicker (9 Hz) responses were also recorded, plotted, and analyzed. Similar dose-dependent functional effects were observed compared to the a- and b-wave amplitudes (see Supplementary Figure S3).

### 2.5. Electroretinographic Recordings Discussed in More Detail in SI-Treated Brown Norway Rats

**Figure 8:**
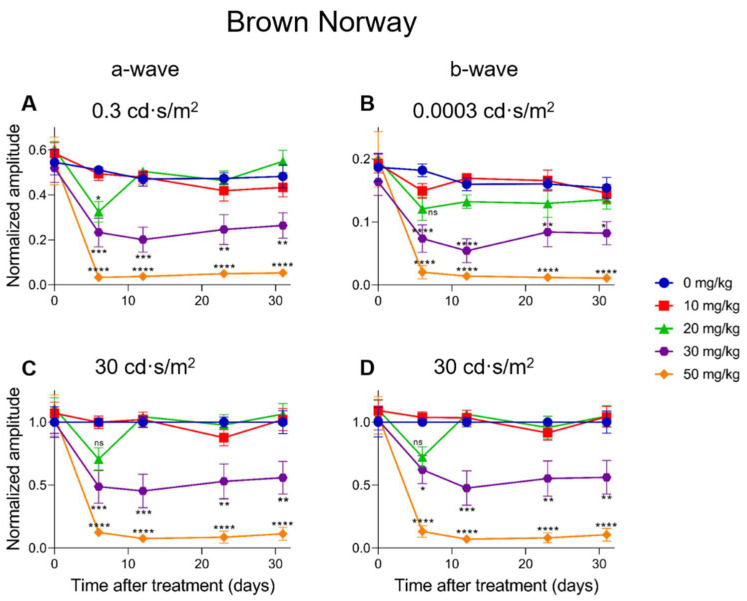
The normalized a-wave (**A**,**C**) and b-wave (**B**,**D**) amplitudes are plotted versus the time after treatment for all treatment groups in Brown Norway rats (*n* = 4 per group). The amplitudes are shown measured at 0.3 cd·s/m^2^ and 30 cd·s/m^2^ (a-wave) and 0.0003 cd·s/m^2^ and 30 cd·s/m^2^ (b-wave) including the standard deviations. No significant effect was observed for the 10 mg/kg group compared to the control. Although not always significant, a temporary effect and significant for the a-wave amplitude at 0.3 cd·s/m^2^ was seen for the 20 mg/kg treatment group. This effect was not observed anymore later in the experiment. From seven-days post-injection onwards, significantly decreased responses were observed for all the other treatment groups. The effects of the 30 mg/kg group were moderate for both the a- and b-wave amplitudes at all light intensities. A tremendous effect was seen for the higher treatment group (50 mg/kg). From one-week post-injection onwards, the recorded a- and b-wave amplitudes decreased to less than 10% of the original value. ns: not significant, *: *p* ≤ 0.05, **: *p* ≤ 0.01, ***: *p* ≤ 0.001, and ****: *p* ≤ 0.0001.

**Figure 9:**
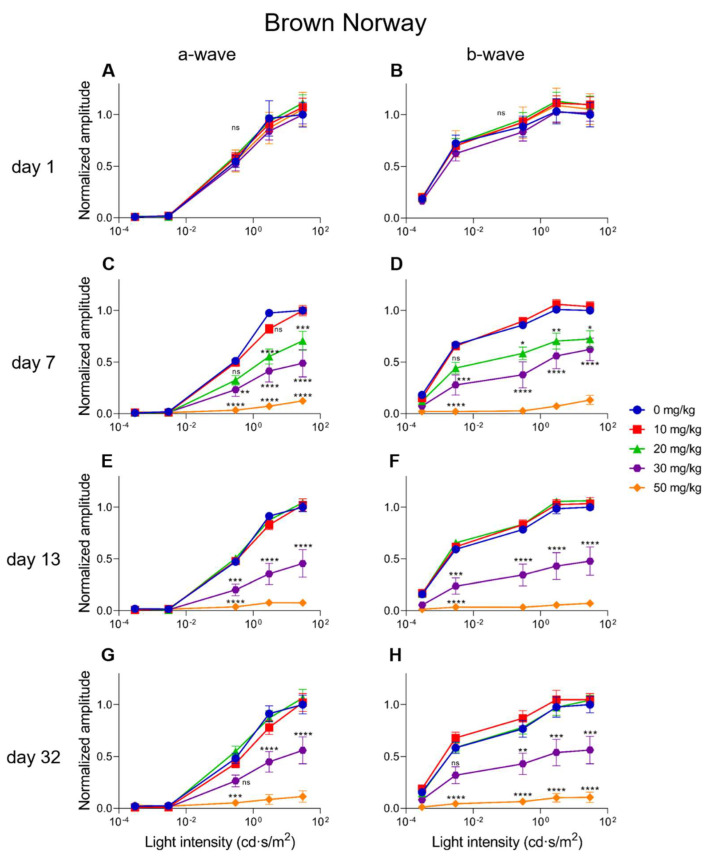
The normalized a-wave (**A**,**C**,**E**,**G**) and b-wave (**B**,**D**,**F**,**H**) amplitudes are plotted versus the light intensity for all the treatment groups in Brown Norway rats (*n* = 4 per group). The amplitudes are shown ± the standard deviations. No significant differences could be observed at day one (baseline) between all the treatment groups. The treatment effects started to show from seven-days post-injection onwards. A significant and clear effect was observed for the 20 mg/kg treatment group for both the a- and b-wave. A 30 mg/kg dose caused moderate effects, and 50 mg/kg caused more extreme decreased amplitudes. From day 13 onwards, more long-term effects were visible. No significant differences were observed (anymore) between the control, 10 mg/kg, and 20 mg/kg groups. A significant, stable, and moderate effect was observed for the 30 mg/kg treatment group. Injection of 30 mg/kg SI caused a reduction of the a- and b-wave amplitudes of roughly 50% at least up to one-month post-injection. More extreme effects were observed for the higher dose (50 mg/kg). Almost non-recordable ERG responses were observed in this group. ns: not significant, *: *p* ≤ 0.05, **: *p* ≤ 0.01, ***: *p* ≤ 0.001, and ****: *p* ≤ 0.0001.3.

A dose-dependent electroretinographic response for SI could also be observed in the Brown Norway rats. A 10 mg/kg SI dose did not cause significant differences compared to the controls (see Figure 8 and Figure 9). A dose of 20 mg/kg caused a significant decrease for the a-wave amplitude one-week post-injection for the low (0.3 cd·s/m^2^) light intensity (Figure 8A). This effect was transient, as it was not seen later in the same experiment. Although drops of the a-wave amplitude at high light intensity (30 cd·s/m^2^) and of the b-wave amplitude both at low (0.0003 cd·s/m^2^) and high (30 cd·s/m^2^) light intensities were observed, it was not significant (Figure 8B–D). Similar to our observations in mice, the 30 mg/kg dose in rats caused a moderate and temporally permanent effect on the ERG responses: Both the a- and the b-wave amplitudes were significantly decreased from one-week post-injection onwards. Reductions of roughly 50–60% were observed. The highest dose (50 mg/kg) in the rats caused a reduction of the a- and b-wave amplitudes to <10% of the original amplitudes. This effect was stable throughout the experiment. Except for the 10 mg/kg treatment group, all the other treatment groups (20, 30, and 50 mg/kg) affected the a- and b-wave amplitudes at seven-days post-injection (Figure 9C,D). At 13-days post-treatment, no differences between the control and the 10 or 20 mg/kg groups were observed (Figure 9E,F). The 30 mg/kg group caused a moderate and stable reduction of the amplitudes for all the light intensities from seven-days post-injection onwards. Almost non-recordable amplitudes were observed for the 50 mg/kg treatment group (Figure 9C–H). Finally, the Flicker (9 Hz) responses were also plotted and analyzed for the Brown Norway rats. Similar dose-dependent functional effects were observed compared to the a- and b-wave amplitudes (see Supplementary Figure S4).

## 3. Discussion

Although numerous rodent models have been developed for (dry) AMD, until now, there is no animal model that is capable of fully representing the (progression of the) dry AMD disease phenotype. At the same time, a number of key mechanistic features of AMD, such as oxidative RPE stress and it consequences, resemble generalized dysfunction of the RPE or even normal physiological aging. Rodents, as also used in this study, share the overall retinal structure with humans and are genetically closely related [60]. A number of genetically-modified models for monogenic retinal degenerations may only represent part of the AMD phenotype [49,60,61,62]. The multifactorial nature of AMD complicates modeling its phenotype considerably [17,63]. Consequently, this lack of representative models severely hampers studies into the etiology and effective treatment modalities of the disease [17,44,63].

In this study, we generated and phenotyped two chemically-induced rodent models for retinal degeneration. C57BL/6J mice and Brown Norway rats were systemically injected intravenously with a wide range of SI doses (0, 10, 20, 30, 40, 50, and 70 mg/kg). They were followed up non-invasively over time by SLO-OCT and ERG. We observed a clear dose-dependent effect on the retinal structural integrity as judged from the OCT images and on the retinal function as judged from the ERG. In summary, we observed that the lower SI doses (0, 10, and 20 mg/kg) did not cause any long-term permanent effects, 30 mg/kg caused moderate effects on retinal structure and function, and higher doses (40, 50, and 70 mg/kg) caused extensive retinal degeneration and almost non-recordable ERG responses. We observed a dose-dependent effect as judged from the ERG data at earlier time points. Later in the experiment, the effects were similar for the higher doses (40, 50, and 70 mg/kg). Although 20 mg/kg did not result in any visible structural retinal damage, DNA fragmentation has been observed in the RPE layer by Koh et al. [64]. This may be functionally consistent with the temporary, although not always significant, quantitative drop that was observed in our ERG responses at the same dose. Indeed, a lower dose of SI may temporarily impact RPE functionality and survival. However, a 20 mg/kg dose is apparently not high enough to contribute to any long-term retinal degeneration. We observed overall similar effects in both species, even though the mice were older than the rats at the start of the experiment (3–4-months versus 8-weeks respectively). Although we cannot exclude an age effect influencing the results, we do not expect a tremendous difference in the outcome if we had included younger mice (e.g., 8-week-old) in our study. Similar results were obtained by Moriguchi et al., who included 8–12-week-old mice [65].

The SI-induced model is a widely used model for retinal degeneration and, more specifically, for AMD [48,52,66,67,68,69]. The model is, for example, used to study the efficacy of RPE transplantations for the treatment of retinal degeneration [59]. However, previously published studies are hard to compare due to differences in the experimental setup, species, strain, dose, administration route, and outcome parameters that have been used and studied [59]. Rodents, especially mice and rats, were most often used in these studies.

Among the mice studies, SI-based experiments have been published involving non-pigmented BALB/c mice and pigmented C57BL/6(J). The administration was usually done intravenously (tail vein, retro-orbital vein) or intraperitoneally. In these studies, the follow-up time varied between three-days and four-weeks, and most read-outs were histological. The SI doses varied between 10 and 50 mg/kg, and the higher doses (>30 mg/kg) were most frequently used [52,55,67,69,70,71]. SI injections in the retro-orbital vein resulted in quick and severe RPE and retinal damage, probably due to its proximity to the retina. For example, Wang and colleagues observed retinal degeneration when using a dose of 20 mg/kg based on histological data at eight-days post-injection [71]. There are some drawbacks to using the retro-orbital vein in experiments involving the eye. For example, anesthesia is always required, and swelling or injury of the eye may occur due to the injection. Since the eye is the subject of the study, in these cases, the animals need to be excluded from the study. In contrast, intravenous injections via the more distant tail vein require no anesthesia, a similar distribution is obtained, and there is no risk for eye damage due to the injection itself [72,73]. We and others did not observe structural damage at 20 mg/kg as measured by OCT. However, the affected ERG amplitudes at this time point do suggest lowered retinal functionality, also in our data. In both the aforementioned datasets, SI injections of 10 mg/kg did not cause any detectable effect [71]. Overall, our results are comparable to those that were obtained in other studies using pigmented mice.

To our knowledge, SI has only been used a few times before in Brown Norway rats [74,75,76]. Relatively high SI doses (35 and 40 mg/kg) and two different administration routes were used in these studies (intravenously and subretinally). An in-depth study giving an overview on the effect of a wide range of SI doses was, to our knowledge, lacking. The systemic intravenous injection caused an overall retinal degeneration in Brown Norway rats, comparable to our results at similar SI concentrations [74,75]. A subretinal injection with SI caused a local effect only [76]. Other rat strains have been used more extensively, such as the non-pigmented strains Wistar and Sprague Dawley and the pigmented strain Long Evans (pigmented hooded). Administration of SI was done intravenously (tail vein or retro-orbital vein), subretinally, or intraperitoneally. The follow-up times in these studies ranged from 1-week to 3-months, and doses varied from 25 to 75 mg/kg. A dose of 50 mg/kg was administered most [70,77,78,79,80].

A comparable study to our setup was performed using the non-pigmented Sprague Dawley strain by Koh et al. [64]. These rats were intravenously injected with 20, 40, 60, and 80 mg/kg SI and followed by ERG up to 10-days post-injection. Retinal morphology was determined by histology, at one time point only: 11-days post-injection. The results using the Sprague Dawley strain were similar to those in our study: little to no retinal damaging effect was observed for the 20 mg/kg dose and more prominent effects for 40 mg/kg and higher, as judged from histology. Unfortunately, the 30 mg/kg dose, which yielded the most interesting transient effect in our study, was not analyzed in the Sprague Dawley strain. Furthermore, little to no differences with our data were observed in the ERG responses between the highest doses: their experiment lasted 11-days, and a dose-dependent effect on the retinal morphology was observed. Additionally, more extensive damage was seen when a higher SI dose was used. Although OCT data is lacking in this study [64], the histological data are consistent with our findings in our in vivo OCT studies.

Administration of a >50 mg/kg SI dose affects the animals’ general health and causes (temporarily) weight loss and doses of 100 mg/kg have been found to be lethal [64,81]. In the eye, the first known retinal cell layer to be affected by SI is the RPE. The RPE is extremely important for the normal function of the retina, as indicated by its role in many retinal degenerative diseases [7,43,44,82,83,84,85]. Severe damage to the RPE layer disrupts the blood-retina barrier and leads to impaired RPE-specific function. As the RPE-photoreceptor complex forms a single functional unit, impairment of the RPE leads to subsequent photoreceptor cell death. This secondary effect was also shown in our previous work reporting on a newly developed genetic rat model for retinitis pigmentosa (*Lrat*^-/-^) [62].

Indeed, other layers of the retina besides the RPE are also eventually affected by SI. In our experiments, we observed thinning of the entire retina. This observation is in line with the observations by others who found SI-induced cell death among the photoreceptors, retinal ganglion cells, and bipolar cells [52,64,67]. Although a direct damaging effect of the systemic sodium iodate on the neural retina cannot be excluded, the most likely scenario is that higher doses of SI contribute to secondary cell death of neural retinal layers as a result of the initial damage to the RPE [64]. Indeed, it would be interesting to look into the sequence of damaging events in the retina. In our study, we were not able to make a definite statement about this. According to our OCT scans, the layers were disrupted due to SI administration. We could not make clear distinctions between the retinal layers in the eyes of the higher treatment groups at the later time points and quantify the degeneration per retinal layer.

Not only the RPE and the retina change with age. Also, the choroid and its vasculature undergo various changes during normal aging. These changes are more prominent and severe in AMD eyes [2]. As the main source of oxygen and nutrients for the outer retina, the choriocapillaris are essential for the survival of the photoreceptor cells and the RPE. It is known that, during normal aging, the choroid thins over time [86]. Moreover, more extensive thinning is observed in early AMD eyes and even more extreme thinning is observed in eyes with geographic atrophy, the endstage of (dry) AMD [87]. Age-related changes to the choroid (and the BrM) may have significant roles in the development of AMD, as many of the changes in these tissues are more dramatic in diseased eyes [88]. The choriocapillaris might be affected even before RPE or photoreceptor cell loss [86,87,88,89]. Indeed, it has been shown in several animal models that SI not only induces RPE and retinal degeneration but also affects the choroid. Although it seems that it is not affecting the choriocapillaris directly, but rather via the RPE. No evidence was observed so far that the choriocapillaris degenerate over patches of healthy RPE [90,91]; only where RPE loss was observed and patches of scar tissue occurred, choriocapillaris atrophy was observed too. This observation led to the hypothesis that the choroid and its choriocapillaris depend on an intact and functioning RPE for its survival [90,91,92,93,94,95,96].

Apart from pigmented models, albino animals have also been widely used in SI studies in mice and rats. It has been postulated that SI reacts with melanin, increasing the conversion of glycine into glucoxylate, a potentially cell toxic compound [52,97]. Interestingly, although albino animals lack melanin, the effects of SI in albino animals seem to be more pronounced. The most dramatic effects were observed using the (Han) Wistar rat [69,70]. Indeed, it appears that the albino retina is more susceptible to SI damage, especially in the central area of the retina where the light is focused [70,98]. Melanin pigment in the RPE has been implicated in protecting the entire retina, including the macula, from oxidative damage [16]. Melanin absorbs excess light and is a (co-)scavenger of oxidative stress-mediated free radicals [99,100]. In albino animals, the melanin pigment is absent. Also, a decline in pigment density is part of normal aging. As a result, the risk of developing AMD becomes higher with older age. Also, less pigmented human individuals may have an increased risk of developing AMD [101,102]. Although an overall comparable effect is reached in both pigmented and non-pigmented rodents, the choice of the model should be carefully made considering possible translational outcomes. Obviously, the majority of the human population has a pigmented RPE layer. This study gives an in-depth overview of the characteristics of the SI-induced model for retinal degeneration in pigmented rodents.

As discussed above, there are many similarities between dry AMD pathology and the effects of SI. Extreme RPE loss can be obtained depending on the dose, similar to geographic atrophy observed in late-stage AMD eyes. Additionally, not only the RPE but also the (outer) retina, the choroidal capillaries and BrM are affected by SI [76]. Disease-related changes to these tissues have been observed in AMD eyes as well. Moreover, the mechanisms that are involved in SI-induced degeneration and atrophy (e.g., oxidative stress and melanin-related pathways) are also known to be involved in AMD pathology and development [5,26]. Further in-depth studies using these models will shed light on the dose-dependency and time windows of the cell types and tissues that are affected by SI, and its relationship with AMD.

In conclusion, SI-induced retinal degeneration in C57BL6/J mice and Brown Norway rats is relatively easy to establish and yields a reproducible model for retinal degenerative diseases, such as dry AMD. The SI-induced retinal degeneration was followed and characterized in depth using OCT and ERG measurements, yielding a detailed insight into the dose-effect of SI over time. This study demonstrates an SI-dose-dependent effect on the retina in both pigmented rat and mouse species; thus, the SI concentration can be selected to generate numerous disease states. No general model for the full disease spectrum of AMD exists yet. The SI-induced model that is presented in this work could be combined with other theorized AMD precursors (e.g., genetic modifications and environmental factors) to identify the critical stages during the evolution of the disease directly. Thus, our data are useful for identifying a therapeutic window and developing experimental therapies for AMD and other retinal degenerative diseases.

## 4. Materials and Methods

### 4.1. Animals

C57BL/6J mice were purchased at the Jackson Laboratory, and Brown Norway rats were purchased at Charles River. All the animals were kept on a light cycle of 12 h on/12 h off and were fed ad libitum. These studies were conducted in accordance with the ARVO Statement for the use of Animals in Ophthalmic and Vision Research and approved by the Netherlands’ national animal welfare committee (AVD1140020172044). Both males and females were used in the mouse studies. Given the availability of animals at the time of purchase, we only used male rats. There is no evidence in the literature that sex difference has a major influence on RPE damage in the context of our studies. Still, it can be considered a possible study limitation. Mice were 3–4-months old at the start of the experiment, weighing 17–20 g, whereas rats were 8-weeks old, weighing 230–260 g.

### 4.2. Sodium Iodate Treatment

Sodium iodate (SI) (Sigma-Aldrich, Hamburg, Germany, CAS number 7681-55-2, NaIO_3_) was freshly diluted in 0.9% sodium chloride (NaCl) into different doses and filter-sterilized directly before use. Electroretinography (ERG), scanning laser ophthalmoscopy (SLO), and spectral-domain optical coherence tomography (OCT) baseline measurements were recorded before the treatment. Subsequent to the baseline measurements, the animals were kept on a heating pad, and the tail vein was stimulated by a heating lamp. The animals were injected into the tail vein using a series of NaIO_3_ doses in 100 µL 0.9% NaCl, see Supplementary Table S1. The animals were randomly divided over the groups (using the tool at www.graphpad.com (accessed on 12 October 2017)).

### 4.3. Visual Follow-Up and Drop-Outs

The animals’ retinal structure and visual function were followed over time using ERG and SLO-OCT measurements. The measurements were performed at day 1 (baseline), 1-week, 2-weeks, 3-weeks, 1-month, and 2-months post-injection. Daily health checks were done, and the weight was measured at least weekly. There were no drop-outs in the group of mice. A total of two rats received a dose of 70 mg/kg and were euthanized, according to the guidelines set in the license (AVD1140020172044), because their weight loss was more than 20% within 3-days. They were excluded from the study.

### 4.4. Electroretinography

The animals were kept in total darkness for at least 2 h before the scotopic measurements and were anesthetized with a mixture of ketamine (65 mg/kg for rats; 100 mg/kg for mice) and xylazine (7.5 mg/kg for rats; 10 mg/kg for mice) intraperitoneally. The eyes were locally anesthetized using tetracaine-hydrochloride drops (1% *w*/*v*) and were dilated using tropicamide (0.5% *w*/*v*) and atropine (1% *w*/*v*) drops. Hylocomod drops were applied to maintain corneal hydration at all times. From this moment onwards, the eye was kept moist using Hylocomod drops that were applied regularly on the animal’s eye. The animals were placed in the RETImap full flash Ganzfeld (Roland Consult, Brandenburg, Germany) using a carrier table kept at 37 °C. The body temperature was carefully monitored during all the measurements. ERGs were recorded using a gold wire loop which was placed on the cornea of both eyes. A gold electrode was placed in the mouth serving as a reference electrode for both eyes. A subcutaneous needle near the tail served as a ground electrode. See Supplementary Table S2 for the light intensities, the number of traces that were used for averaging, and the flashes’ interval. The ERG traces were 350 ms long utilizing 512 data points.

All the data were systematically analyzed, without human intervention, using a custom Matlab script. The data were zero-centered by averaging the signal before the stimulus (<20 ms) and subtracting the resultant from the entire trace. A low-pass filter (4th order, 30 Hz (for the b-wave) and 235 Hz (for the a-wave)) was applied, in both the forward and backward direction, to remove noise and the oscillatory potentials (OPs) without phase-shifting the time-series data. The frequency 30 Hz is well below the minimum expected frequency, and 235 Hz resembles the expected maximum frequency of Ops in rats [103]. The *findpeaks* function in Matlab was used to find the latencies of the a- and b-waves in the filtered data. The magnitudes of the unfiltered signal at the selected latencies were characterized as the values for the b-wave and the absolute a-wave amplitude. The absolute a-wave was subtracted from the value of the b-wave amplitude to calculate the absolute b-wave amplitude. The Flicker properties were determined from the original, unfiltered trace, see Supplementary Figure S1. The time to the first peak (P1) and the amplitude of the second peak (P2) were identified. The b-wave, a-wave and flicker properties of each group, at each treatment day, were averaged and normalized to the corresponding 30 cd·s/m^2^ response from the control group (see Supplementary Figure S2).

### 4.5. Scanning Laser Ophthalmoscopy (SLO) and Optical Coherence Tomography (OCT)

The SLO and OCT measurements were done subsequently to the ERG measurements. Detailed methods are described elsewhere [104,105]. In short, we used a commercially available system (Spectralis^®^ Heidelberg Engineering combined, Heidelberg, Germany) that was modified for use with animals (Medical Workshop, Groningen, The Netherlands). After ERG measurements, the animals received another tetracaine hydrochloride eye drop, and a contact lens (2.7 mm in diameter for mice, 5.2 mm in diameter for rats; Cantor-Nissel, Brackley, UK) was placed. The standard 30° field of view equipment set was used. The animals were placed on a custom-made heated holder, the eyes were kept moist using Hylocomod eye drops (Ursapharm, Saarland, Germany), and the body temperature was monitored. Imaging was done using the Eye Explorer software version 1.9.14.0 (Heidelberg Engineering, Heidelberg, Germany). For fundus imaging (infra-red reflection SLO), the intensity was adjusted to prevent overexposure. OCT imaging was performed using a volume scan (57 frames (ART), 786 A-scans, 30° × 25°, 61 scans, Δ120 µm, 8.8 scans per second). The reference arm was adjusted according to the manufacturer’s instructions. Whenever possible, the follow-up function was used to ensure the accurate thickness profiles between the time points. Frame analysis was done on a total of 5 single OCT scans and the corresponding thickness profiles. The chosen scans were the crossing of the optic nerve (ON = 0), the middle superior section (ON + 10), the superior section (ON + 20), the middle inferior section (ON − 10), and the inferior section (ON − 20). The total retinal thickness was determined within each selected OCT scan with 1 mm intervals, with a 0.5 mm minimum distance from the optic nerve. During recovery, the eyes were treated with Vidisic ^®^ Carbogel eyegel (Bausch + Lomb; Brussel, Belgium).

### 4.6. Statistical Analyses Performed on OCT and ERG Data

The data were analyzed using one- or two-way ANOVA analyses, ANOVA analyses of the log-transformed data, and the Kruskal–Wallis analyses with a post hoc Bonferroni test to determine the statistical significance of all data. Similar *p*-values were obtained using all tests. *p*-values are reported: ns: not significant, *: *p* ≤ 0.05, **: *p* ≤ 0.01, ***: *p* ≤ 0.001, and ****: *p* ≤ 0.0001.

## Data Availability

The data presented in this study are available on request from the corresponding author.

## Supplementary Materials

The following supporting information can be downloaded at: https://www.mdpi.com/article/10.3390/ijms23062918/s1.

## Abbreviations

ABCA1	ATP-binding cassette subfamily A member 1
AMD	Age-related macular degeneration
ANOVA	Analysis of variance
APOE	Apolipoprotein E
ARMS2	Age-related maculopathy susceptibility 2
ATP	Adenosine tri-phosphate
BrM	Bruch’s Membrane
C9	Complement C9
CFH	Complement Factor H
CFI	Complement Factor I
COL15	Collagen type XV alpha 1 chain
COL8A1	Collagen type VIII alpha 1 chain
ERG	Electroretinography
GPX4	Glutathion peroxidase 4
HTRA1	High-temperature requirement A serine peptidase 1
i.v.	Intravenous
kb	Kilobases
Lrat	Lecithin:retinol acetyltransferase
MMP19	Matrix metallopeptidase 19
MMP9	Matrix metallopeptidase 9
OCT	Optical coherence tomography
ON	Optic nerve
OP	Oscillatory potentials
PLTP	Phospholipid transfer protein
ROS	Reactive oxygen species
RPE	Retinal pigment epithelium
SI	Sodium iodate
SLO	Scanning Laser Ophthalmoscopy
TIMP3	Tissue inhibitor of matrix metalloproteinases 3
VTN	Vitronectin
