# Supplementary material for: Sodium-Iodate Injection Can Replicate Retinal Degenerative Disease Stages in Pigmented Mice and Rats: Non-Invasive Follow-Up Using OCT and ERG
Source: Int J Mol Sci. 2022 Mar 8;23(6):2918. doi: 10.3390/ijms23062918 (PMC8953416; doi:10.3390/ijms23062918)
Supplement: Supplementary file 1 [file ijms-23-02918-s001.zip › ijms-1605634-supplementary.pdf]

Supplementary Materials

Supplementary Table S1. An overview of the experimental groups.

Species	Strain	Number of animals	Treatment
Mouse	C57BL/6J	4	0.9% NaCl
		4	10 mg/kg
		4	20 mg/kg
		4	30 mg/kg
		4	40 mg/kg
		4	50 mg/kg
		4	70 mg/kg
Rat	Brown Norway	4	0.9% NaCl
		4	10 mg/kg
		4	20 mg/kg
		4	30 mg/kg
		4	50 mg/kg
		2	70 mg/kg

Supplementary Table S2. An overview of the settings for electroretinography measurements.

Intensity (cd/m^2)	Averaged	Interval (Hz)
0.0003	20	0.2
0.003	18	0.125
0.03	14	9 (Flicker)
0.3	15	0.077
3	12	0.067
10	8	0.05
30	8	0.05

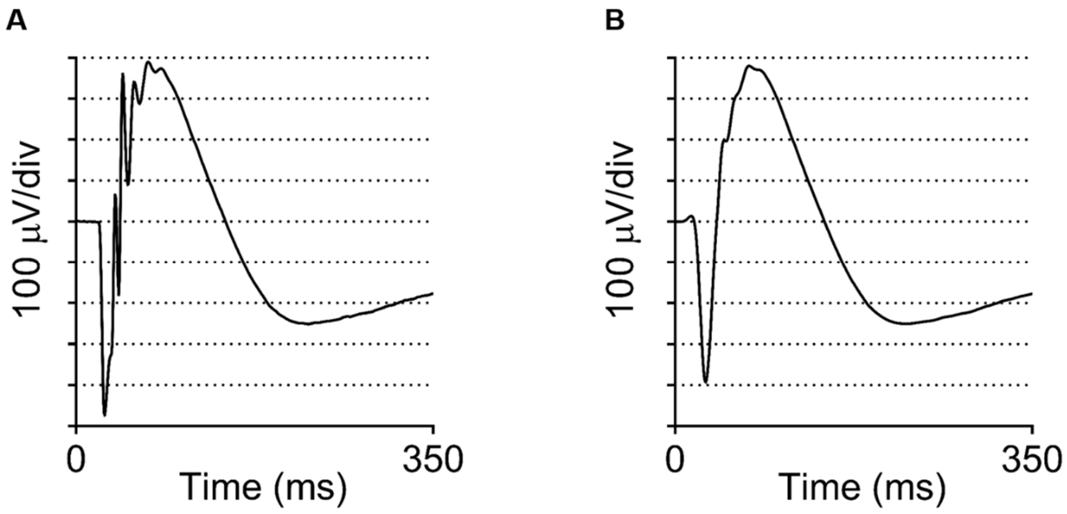

Supplementary Figure S1. An example of an ERG trace before (A) and after (B) data filtering is shown. The trace belongs to an untreated Brown Norway rat from which the ERG is measured at 30 cd·s/m^2. The oscillatory potentials are filtered out to determine the latencies of the a- and b-wave (in B). Subsequently, the latencies are used to determine the values of the amplitudes in the unfiltered data (A).

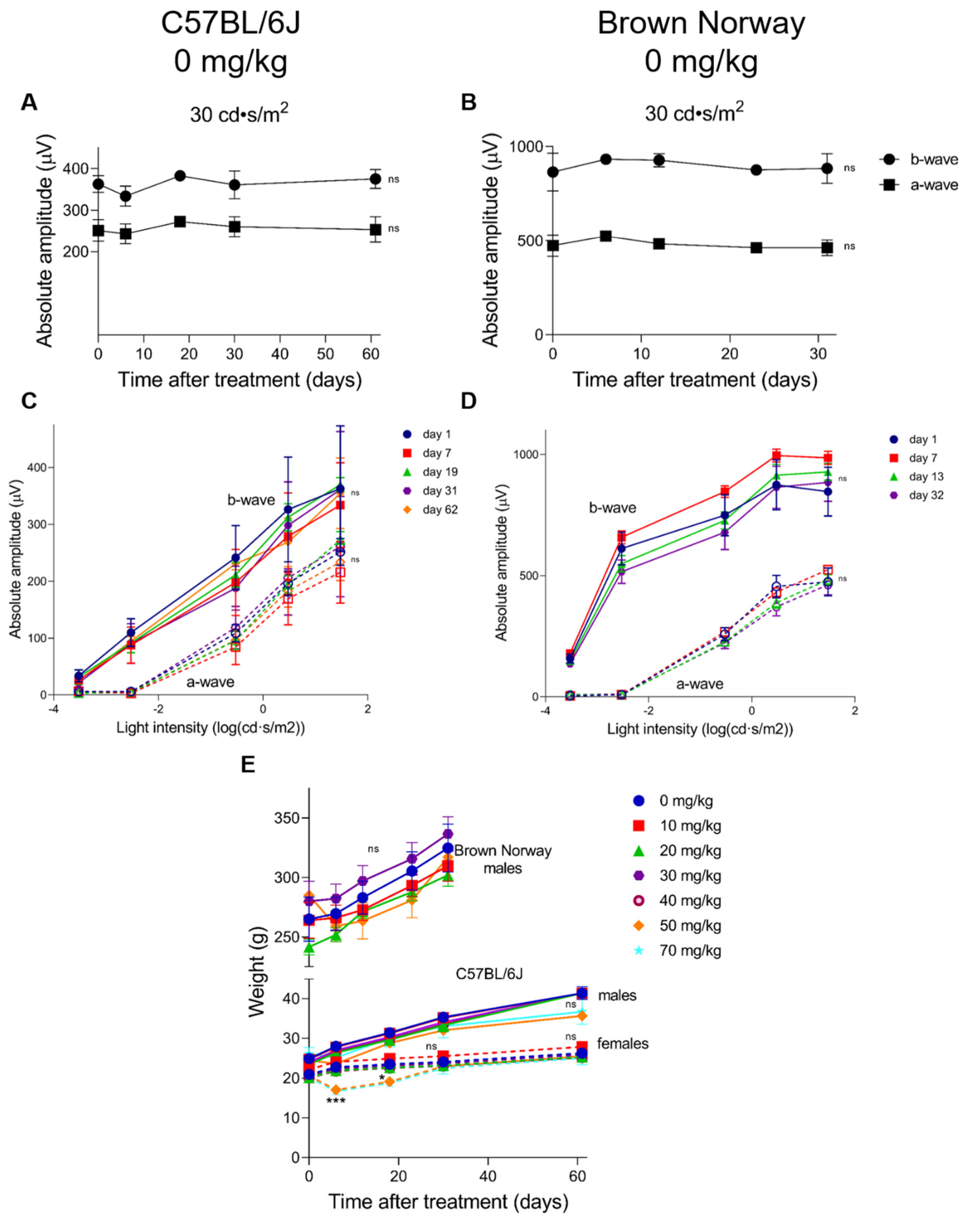

Supplementary Figure S2. The absolute values for the a- and b-wave amplitudes at the highest light intensity (30 cd·s/m²) over time (A and B) and versus the light intensities (C and D) are shown for both mice (A and C) and rats (B and D) (n = 4 per group). No significant differences can be observed over time within the control groups (0 mg/kg SI) for both species. In E, the weight progression of both rats and mice is shown. Within the group of mice, a significant drop in weight progression is seen for the highest doses (50 and 70 mg/kg). At 3 weeks post-injection, no difference can be observed between all groups of mice. For the rats, there is no significant difference in weight progression visible. However, the 70 mg/kg group (n=2) was excluded from the study since they lost >20% of their weight within 3 days post-injection, reaching a humane endpoint. All

data points are plotted with standard deviations (although not always visible in the graph). If not visible, it means that their value is not larger than the symbol that is used in the graph. *ns*: not significant.

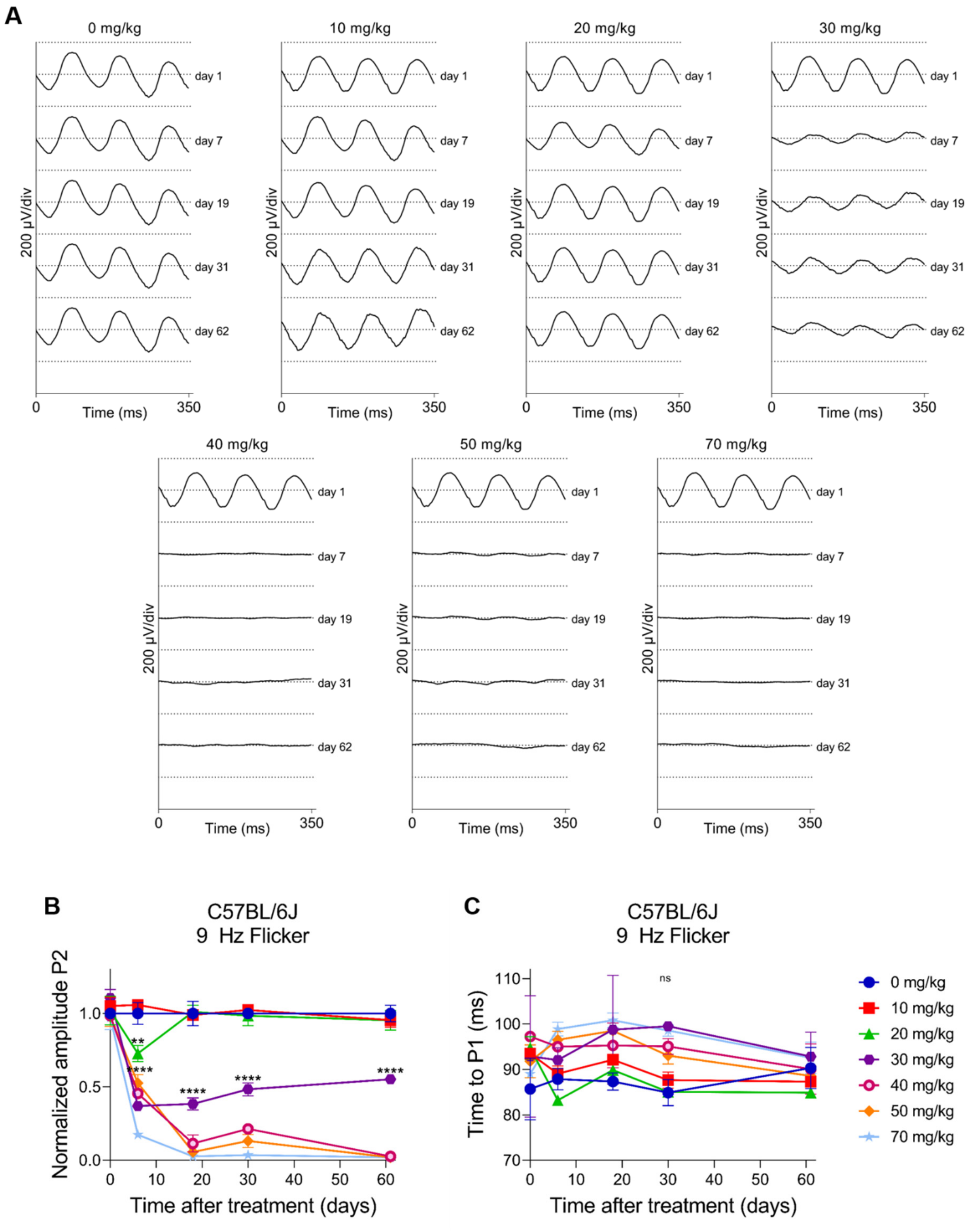

Supplementary Figure S3. ERG Flicker data (9 Hz) is shown for C57BL/6J mice (*n* = 4 per group). From the traces (A), it is clear that by eye, there is no clear difference between the 10 mg/kg group and the control group (0 mg/kg). A slight effect of SI-treatment is seen for the 20 mg/kg group after a week. However, this effect is gone after two weeks. Moderate effects of SI-treatment are seen for the 30 mg/kg group already within a week post-injection. This effect seems stable over time. The tremendous impact is seen for the higher doses (40, 50 and 70 mg/kg). Flicker responses are (almost) completely absent

already within a week after injection. These observations were confirmed by quantifying the data (B and C): No significant differences were observed between the 10 mg/kg group and the control group. A significant drop in P2 amplitude was observed for the 20 mg/kg group. A quick and moderate effect is seen for the 30 mg/kg group, with no significant difference between the last two time points. The higher doses (40, 50 and 70 mg/kg) cause an immediate and tremendous effect with hardly any measurable activity from 20 days post-injection onwards. No significant differences were observed for the time to P1 between all treatment groups. *ns:* not significant, **: $p \leq 0.01$, and ****: $p \leq 0.0001$.

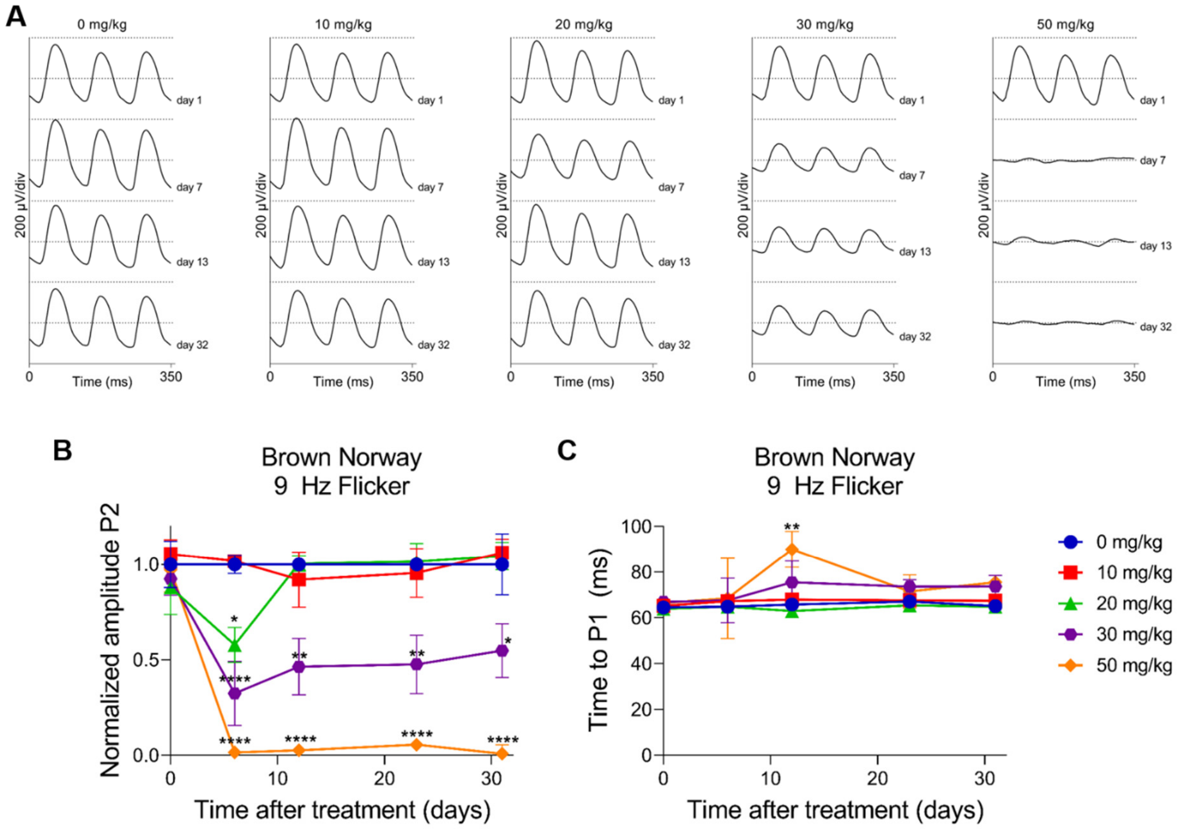

Supplementary Figure S4. ERG Flicker data (9 Hz) is shown for Brown Norway rats (*n* = 4 per group). From the traces (A), it is clear that by eye, there is no clear difference between the 10 mg/kg group and the control group (0 mg/kg). A slight effect of SI-treatment is seen for the 20 mg/kg group after a week. However, this effect is gone after two weeks. Moderate effects of SI-treatment are seen for the 30 mg/kg group already within a week post-injection. This effect seems stable over time. The tremendous impact is seen for the higher dose (50 mg/kg). Flicker responses are (almost) completely absent already within a week after injection. These observations were confirmed by quantifying the data (B and C): No significant differences were observed between the 10 mg/kg group and the control group. A significant drop in P2 amplitude was observed for the 20 mg/kg group. A quick and moderate effect is seen for the 30 mg/kg group, with no significant difference between the last three time points. The higher dose (50 mg/kg) causes an immediate and tremendous effect with hardly any measurable activity from already 7 days post-injection onwards. A significant difference was observed for the time to P1 between the 50 mg/kg group and the control group. However, it is debatable whether this parameter could be observed properly within this dataset. No other significant differences could be observed between the other groups. *ns*: not significant, *: $p \leq 0.05$, **: $p \leq 0.01$, and ****: $p \leq 0.0001$.
